# Experience-based food insecurity in Bangladesh: Evidence from Household Income and Expenditure Survey 2022

**DOI:** 10.1016/j.heliyon.2024.e41581

**Published:** 2025-01-06

**Authors:** Faria Rauf Ria, Md. Muhitul Alam, Md. Azad Uddin, Mohaimen Mansur, Md. Israt Rayhan

**Affiliations:** a Institute of Statistical Research and Training, University of Dhaka, Bangladesh; b Bangladesh Bank, Bangladesh

**Keywords:** Food insecurity, Food insecurity experience scale, Rasch model, Multilevel logistic regression, Classification tree, Variable importance, Bangladesh

## Abstract

This paper examines the current state of food insecurity in Bangladesh and its socio-economic drivers using data from the latest Household Income and Expenditure Survey (HIES 2022). Unlike previous studies that relied on less precise measures of food insecurity, such as food expenditure, diversity, and calorie intake, this study employs the internationally recognized Food Insecurity Experience Scale (FIES) and Rasch model-based thresholds to classify households as food secure or insecure. Multilevel logistic regression is used to identify significant predictors of moderate and severe food insecurity, considering the hierarchical structure of the data, with households nested within geographical clusters. Key factors found to be significantly associated with food security include the wealth index, land ownership, education of the household head, family size, remittance income and exposure to shocks. A classification tree, a popular machine learning method, is also applied to explore important interactions among these determinants. The tree analysis confirms the importance of several regression-based predictors and identifies households at the highest risk of food insecurity through variable interactions. Factors such as poverty, lack of land ownership, low education levels, and high dependency ratios collectively increase a household's vulnerability to moderate food insecurity to around 51% while the national prevalence is 19%. District-level maps of food insecurity prevalence reveal significant regional disparities, underscoring the need for targeted, district-specific interventions to effectively combat food insecurity. More broadly, policies promoting education and family planning, training in better shock management, and facilitating remittance flows through simplified processes may contribute to addressing the food insecurity challenge.

## 1 Introduction

Food insecurity has become a significant global concern, affecting both developed and developing nations. Under the Sustainable Development Goal (SDG) 2, all United Nations member states have pledged to eradicate hunger and improve nutrition by 2030 [1]. However, approximately 735 million people still face hunger daily, with varying levels of severity, and around 2.4 billion people worldwide experience moderate or severe food insecurity, making it a critical global issue. The highest proportion of the population experiencing hunger is in Africa at 20%, followed by Asia at 8.5% [2]. According to the United Nations Food and Agriculture Organization (FAO), food insecurity is defined as a lack of regular access to sufficient safe and nutritious food for normal growth, development, and an active, healthy life. Food insecurity is a multi-dimensional issue, encompassing key aspects such as food availability, accessibility, utilization, and stability.

The consequences of food insecurity extend far beyond hunger and malnutrition [3]. Household food insecurity is a major factor affecting maternal health and child nutrition, compromised immune systems, developmental delays, poor academic performance, and mental and physical health issues among adults, including chronic conditions like diabetes and heart disease, which can have long-term effects on health and productivity [4], [5]. Food insecurity disproportionately impacts people living in rural areas, where residents are at a higher risk of experiencing food insecurity [6]. Additionally, food insecurity can exacerbate conflicts and, in some cases, lead to migration [7], [8].

Academic research provides valuable insights into the regional variations, cultural contexts, and geopolitical factors that influence food access and security globally. Economically vulnerable populations are particularly at risk of food insecurity, as they often face numerous financial barriers limiting their access to sufficient, safe, and nutritious food [9]. From a macroeconomic perspective, factors such as economic slowdowns, inflation, unemployment [10], armed conflicts, institutional and political instability [11], and climate-related events such as droughts, floods, and hurricanes [12], are considered as the major drivers of food insecurity. From a microeconomic standpoint, household factors such as family size, composition, access to resources, low education levels, and limited social capital increase the risk of food insecurity. The COVID-19 pandemic posed unprecedented challenges to food security, significantly disrupting food access and increasing household food insecurity by nearly one-third in some regions, as seen in a study from the United States [13].

Food insecurity remains one of the most burning issues facing Bangladesh. Despite remarkable economic growth and success in reducing poverty, hunger, and improving food security over the past decades [14], food insecurity continues to affect the livelihoods and well-being of millions [15]. With approximately 169.8 million people living in a relatively small geographic area [16], Bangladesh has one of the highest population densities in the world. Rapid population growth puts immense pressure on the country's food supply and agricultural resources, exacerbating food security challenges. According to the Global Hunger Index 2023, Bangladesh is classified as having “moderate” hunger levels [17].

Over the years, the Bangladesh government and various non-governmental organizations (NGOs) have worked extensively to improve agricultural productivity, diversify food sources, and implement social safety nets to mitigate food insecurity risks [18]. Bangladesh also receives support from international organizations such as the World Food Programme (WFP) to enhance food security and nutrition. Chronic food insecurity is particularly prevalent in rural areas, where agricultural productivity is low and income opportunities are limited [19]. Acute food insecurity, often temporary, usually occurs due to sudden shocks such as natural disasters, economic crises, or conflicts. Bangladesh, being highly susceptible to natural disasters like floods, cyclones, and droughts, frequently experiences bouts of acute food insecurity [19], [20].

Experience-based food insecurity measures are critical for assessing access to food. The Food Insecurity Experience Scale (FIES), developed by the FAO's Voices of the Hungry (VoH) project, is one such scale that aims to produce comparable estimates of food insecurity across countries [21]. The FIES significantly enhances food security assessment by more accurately capturing the access dimension of food security. It includes eight dichotomous questions ranging from concerns about food scarcity to experiencing a full day without food due to a lack of money or other resources over a specified period (e.g., 12 months). The FIES operates on the assumption that the severity of food insecurity in a household or individual can be analyzed as a latent trait, which can be inferred from observable evidence using measurement models based on Item Response Theory (IRT). Utilizing the psychometric properties of FIES, the Rasch model, rooted in IRT, is employed to estimate food insecurity status [22]. Several studies have used FIES data to measure household food insecurity globally [23], [24].

While numerous studies have explored consumption-based food insecurity [25], [26], experience-based food insecurity remains a relatively new concept in Bangladesh and has yet to be thoroughly investigated. Most existing research predates the COVID-19 pandemic [27], and none have used the most recent HIES survey conducted post-pandemic. Furthermore, many studies rely on small, region-specific samples rather than nationally representative data [28]. Although identifying key factors associated with food insecurity is common, many studies overlook the inherent clustering of data [29] and fail to explore potential interactions between variables, especially in the context of Bangladesh. Additionally, complex machine learning techniques have not yet been applied to this issue in the country. There is also a notable gap in research on the spatial variation of food insecurity, particularly in recent times, which is crucial for designing region-specific interventions.

This paper aims to identify the key demographic and socio-economic determinants of food security and to determine the high-risk subgroups of food insecurity using the most recent nationally representative Household Income and Expenditure Survey (HIES 2022) data, which incorporates the FIES questions for the first time. It also seeks to examine the regional variation in food insecurity prevalence. By studying food insecurity, researchers can assess its long-term implications on human development, educational attainment, economic productivity, and societal stability, helping to forecast trends and prepare for future challenges related to food security.

Our study leverages the comprehensive HIES 2022 survey, which offers nationally representative data with a substantial sample size. By incorporating the newly introduced FIES data, we delve into experience-based food insecurity. We utilize machine learning techniques, specifically Classification Trees, to analyze the complex interactions between various determinants of food insecurity. This approach allows us to identify specific high-risk subgroups, guiding targeted interventions. Additionally, our use of multilevel logistic regression addresses clustering effects within the data and highlights key determinants of food insecurity, including urban-rural disparities. Finally, we create district-wise maps showing the prevalence of food insecurity, which will support the development of region-specific strategies to combat food insecurity and alleviate hunger.

The rest of the paper is organized as follows: Section 2 discusses the data summary and describes the main variables. Section 3 presents the empirical strategy for our estimation. Section 4 outlines the estimation results, and Section 5 discusses the major findings. Finally, Section 6 draws conclusions and makes policy recommendations.

## 2 Research design and data

### 2.1 Source of data and sample size

This study relied on the data from the most recent Household Income and Expenditure Survey (HIES) 2022 conducted by Bangladesh Bureau of Statistics (BBS), a multi-subject survey covering varied socio-economic features. The survey was administered from 1 January to 31 December 2022, adopting a two-stage stratified cluster sampling technique. The sampling frame for the survey was designed from the available second zonal operation of Population and Housing Census 2022. The eight administrative divisions were categorized into rural and urban areas, resulting in 16 primary domains or strata (8 rural and 8 urban) for the survey. The primary sampling units (PSU) were the enumeration areas (EA) and each EA consisted of 100 households. A total of 720 PSUs were selected, with 45 from each stratum, and 20 households per PSU, resulting in a sample of 14,400 households. A new section to provide data on the FIES was included in HIES 2022. This section contains 8 questions developed by FAO. Our analysis included 14,272 households with complete data on 8 FIES questions. Further details about the survey design and questionnaire can be found in the report of HIES 2022 [30].

### 2.2 Research variables

The three outcome variables considered in the study are moderate or severe food insecurity, moderate food insecurity and severe food insecurity. To obtain the outcome variables, a raw score needs to be calculated for each household. This raw score is computed based on the 8 questions of FIES included in HIES. The 8 questions have been developed by Food and Agricultural Organization (FAO) and is widely accepted and utilized to measure food insecurity around the world [31]. Table 1 contains the 8 questions of the FIES along with the standard levels used to denote these questions.

**Table 1:** Questions of the FIES used in HIES survey.

Item	Item Description (During the last 12 months, was there a time when?)	Label
1	You were worried you would not have enough food to eat because of a lack of money or other resources	WORRIED
2	You were unable to eat healthy and nutritious food because of a lack of money and other resources	HEALTHY
3	You ate only a few kinds of foods because of a lack of money or other resources	FEWFOODS
4	You had to skip a meal because there was not enough money or other resources to get food	SKIPPED
5	You ate less than you thought you should because of a lack of money or other resources	ATELESS
6	Your household ran out of food because of a lack of money or other resources	RUNOUT
7	You were hungry but did not eat because there was not enough money or other resources for food	HUNGRY
8	You went without eating for a whole day because of a lack of money or other resources	WHLDAY

The raw score represents the total number of “yes” answers given across the eight questions. The thresholds given by FAO were used in categorizing the households by severity of food insecurity (FI) [21]. Thus we created three binary variables as: (1) Moderate or severe FI=1 if raw score ≥4, (2) Severe FI=1 if raw score ≥8 and (3) Moderate FI=1 if raw score ≥4 but ≤7. The raw score cutoff were determined based on the adjusted threshold obtained by applying the Rasch model and equating to the FIES global standard scale.

The independent variables considered in the study are selected based on a review of the literature. They include: Area of Residence (Urban, Rural) [6], [15], Electricity Hours [32], Household Head Sex (Male, Female) [27], [28], Household Head Age [15], [28], Household Head Education (No education, Primary, Secondary, Higher) [6], [15], Total Land [33], [34], Safety Net (No, Yes) [35], [36], Household Shock (No, Yes) [4], Presence of Chronic Disease (No, Yes) [37], [38], Wealth Index (Poorest, Poorer, Middle, Richer, Richest) [39], [40], Division (Barishal, Chattogram, Dhaka, Khulna, Mymensingh, Rajshahi, Rangpur, Sylhet) [15], [27], Dependency Ratio [41], [42], Household Members [28], Number of Earners [15], [27] and Received Remittance (No, Yes) [7], [29]. The wealth index variable was created through Principal Component Analysis (PCA). Relevant variables were selected first following the suggestions of Demographic and Health Survey (DHS) and PCA was applied. The scores of the first principal component were retained and grouped into five categories based on the quantiles to obtain the wealth index.

## 3 Methodology

### 3.1 Rasch model

Rasch model, based on IRT is applied in the measurement of food insecurity based on FIES, assuming the severity of food insecurity for a household can be analyzed as a latent attribute [43]. It is an instrument for quantifying unobservable human conditions based on empirical data and has been widely adopted in applied social and health research [31]. The model has an ability parameter $\theta_{h}$ for each person *h* and a difficulty parameter $\beta_{i}$ for each item *i*. The model assumes equal discrimination for all items and responses to the items are independent. The parameters depict the positions of the items and the persons on the shared latent variable (measured on the same one-dimensional scale). It is a probabilistic model that attempts to make a link between the latent trait (unknown food insecurity measure) and the responses (observable dichotomous responses to experience-based questionnaires). It can also be mentioned as the one parameter logistic (1PL) model.

The logistic function of the probability for a respondent facing a certain experience is modeled as the difference between the person and item severity parameters ($\theta_{h}-\beta_{i}$). The model is written as

(1) $$\mathrm{Prob}(x_{h,i}=1|\theta_{h},\beta_{i})=\frac{\exp(\theta_{h}-\beta_{i})}{1+\exp(\theta_{h}-\beta_{i})},$$

where $x_{hi}$ represents the response of the respondent *h* to the item *i* which takes value 1 for “yes” and 0 for “no” in equation (1). Conditional maximum likelihood is used to estimate the parameters. After the estimation of the parameters, equating is done with the global standard scale to obtain the adjusted threshold and the prevalence rate. For identifying the possible determinants regression is necessary and for this purpose each household is classified in one of the food security status based on the raw score. From the properties of Rasch model, the raw score is a sufficient statistic for measuring the latent trait. It refers household with the same raw score will be considered in the same food security status [44].

### 3.2 Classification tree

Machine learning techniques based on trees are often employed to identify nonlinear effects in response variables and to examine interactions between predictor variables [45]. These methods stratifies the covariate space into some regions and predicts the response. In case of predicting qualitative response, classification trees are used. Let $X_{1}$, $X_{2}$, ..., $X_{p}$ be the *p* covariates and the set of possible values for the covariates are divided into *J* distinct and non-overlapping regions $R_{1}$, $R_{2}$, ..., $R_{j}$. The regions are chosen to be high-dimensional rectangles based on the classification error rate defined as

(2) $$E=1-\max_{k}({\overset{ˆ}{p}}_{mk}),$$

where ${\overset{ˆ}{p}}_{mk}$ is the proportion of observations in *m*th region and from *k*th class in the training set in equation (2).

The approach of recursive binary splitting is adopted to build the classification tree. The approach starts at a point where all observations are in a single region (top of the tree) and then splits successfully into two new regions (branches) in each split. More formally, at first two regions are defined for any *j* and *s* as

(3) $$R_{1}(j,s)=\{X|X_{j}<s\}\;\text{and}\;R_{2}(j,s)=\{X|X_{j}\geq s\},$$

where *j* and *s* are chosen based on the classification error rate in equation (3).

In this way the process is repeated and splitting is done in one of the two regions, defined earlier. In our decision tree modeling, we employed specific criteria to manage the complexity and effectiveness of the model. A critical criterion was the complexity parameter, which controls the degree of tree growth by penalizing splits that do not significantly improve predictive accuracy. We also set a minimum requirement of 5 observations per node before further division, ensuring that nodes had adequate data for meaningful splitting [45].

### 3.3 Multilevel logistic regression model

The secondary data used in this study was collected using a stratified cluster sampling technique. When analyzing such data, it is important to account for correlations between observations to accurately estimate standard errors and conduct hypothesis testing. To address the correlation of observations within primary sampling units (PSUs), a random intercept multilevel logistic regression model can be employed. Let *Y* denote the binary response variable and $X_{1},X_{2},\ldots ,X_{p}$ denote *p* covariates, then the random intercept multilevel logistic regression model is defined as

(4) $$\text{logit}(\pi_{ij})=\log\left(\frac{\pi_{ij}}{1-\pi_{ij}}\right)=\beta_{00}+b_{0j}+\beta_{1}X_{1ij}+\beta_{2}X_{2ij}+\ldots +\beta_{p}X_{pij},$$

where $\pi_{ij}$ is the probability that *Y* is equal to 1 for *i*-th individual in *j*-th PSU, $\beta_{0j}=\beta_{00}+b_{0j}$ is the intercept for the *j*-th PSU, which is independent, identically and normally distributed with mean 0 and constant variance $\sigma_{b}^{2}$ [46]. Estimation of the model in equation (4) is challenging due to the non-linearity of the random effects in the joint distribution of *Y* and $\beta_{0j}$. To address this, adaptive Gaussian quadrature is employed to evaluate the integral of $f(Y,\beta_{0j})$ and obtain the marginal distribution of *Y* [47]. For this process, 10 quadrature points are employed to ensure a balance between computational efficiency and accuracy in the estimation.

### 3.4 Ethical approval

This study uses HIES 2022 data which is observational in nature and does not involve any direct interaction with human participants or the collection of personal data. The source of the data is secondary in the sense that data have been obtained from data repository of the Bangladesh Bureau of Statistics (BBS) upon request. All of the respondents are unidentified and oral consent was obtained from each participant prior to the interview. As such, it does not require ethical approval.

## 4 Results

### 4.1 Reliability of FIES from Rasch model

Table 2 contains the item fit statistics and the reliability value of the Rasch model. The item fit statistics evaluate how well each item on the scale aligns with the underlying latent trait by comparing the observed response patterns with those expected by the measurement model. The reliability value is a measure to assess the overall model fit. For all the FIES items, the infit values fall between 0.7 and 1.3 which suggests all items are consistently associated with the latent trait. It also imply that the assumption of discriminating equally is fulfilled. The model shows a satisfactory overall fit, as indicated by a reliability value of 0.79. In case of outfit statistics, the values are considerably small and less than 2 except the item “healthy” which showed a large outfit value. This may be due to a few highly unexpected responses but considering the infit value and the overall reliability, the item is retained in the model.

**Table 2:** Item fit statistics and Rasch reliability.

**Item**	**Infit (SE of Infit)**	**Outfit**	**Reliability**
Worried	1.23 (0.02)	1.38	0.79
Healthy	1.12 (0.01)	22.43	
Fewfood	0.79 (0.02)	0.77	
Skipped	0.88 (0.04)	0.63	
Ateless	0.90 (0.02)	0.82	
Runout	0.87 (0.03)	0.77	
Hungry	0.82 (0.04)	0.60	
Whlday	1.02 (0.13)	1.03	

### 4.2 Exploratory analysis

**Figure 1:**
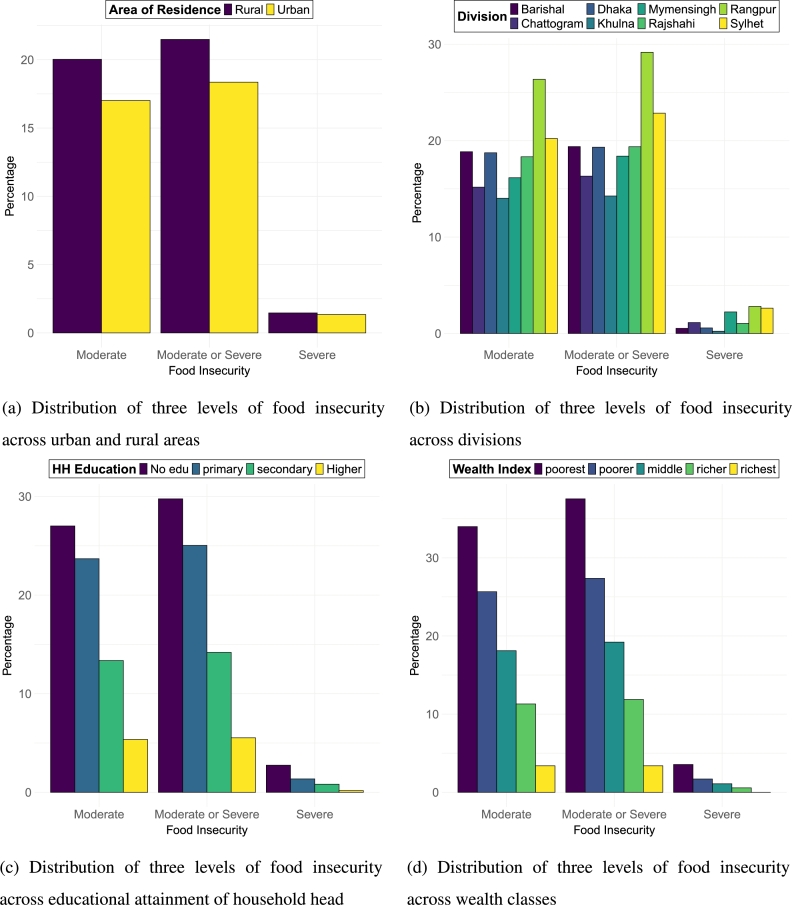
Distribution of food insecurity across various socio-economic dimensions: (a) Distribution of three levels of food insecurity between urban and rural areas. (b) Distribution of three levels of food insecurity across different divisions. (c) Distribution of three levels of food insecurity based on the educational attainment of the household head. (d) Distribution of three levels of food insecurity among different wealth classes.

The nationwide prevalence of the food insecurity status across different levels are 20.49% for moderate or severe food insecurity, 19.24% for moderate food insecurity and 1.25% for severe food insecurity. Fig. 1 shows the distribution of food insecurity across different socio-economic and demographic variables. Households in rural area encounter a higher prevalence of moderate or severe food insecurity which is 21.5% compared to urban households having 18.4%. Also, rural households experience greater severe food insecurity (1.5%) than the urban (1.3%). Rangpur division shows the highest whereas Khulna division has the lowest prevalence of food insecurity in all three levels. Specifically for moderate or severe food insecurity, the prevalence is as high as 29.2% for Rangpur division and 14.3% for Khulna division.

The figure also reveal a clear inverse relationship between the level of education of the household head and the prevalence of food insecurity. Considering severe or moderate food insecurity, households with no educated household head have a prevalence of 29.8% which steadily decreases to only 5.5% with households having higher educated household heads. Likewise, 27% of households suffer from moderate food insecurity and 2.8% of households suffer from severe food insecurity where household heads have no formal education and the percentage drops to just 5.4% and 0.2% with higher educated household heads. Households belonging to different wealth quintiles suffer from a prevalence of moderate or severe food insecurity that varies from a maximum of 37.5% in the poorest quintile to a minimum of 3.4% in the richest quintile. The same pattern is noticed for moderate and severe food insecurities in households of varying wealth quintiles.

### 4.3 Classification tree analysis

**Figure 2:**
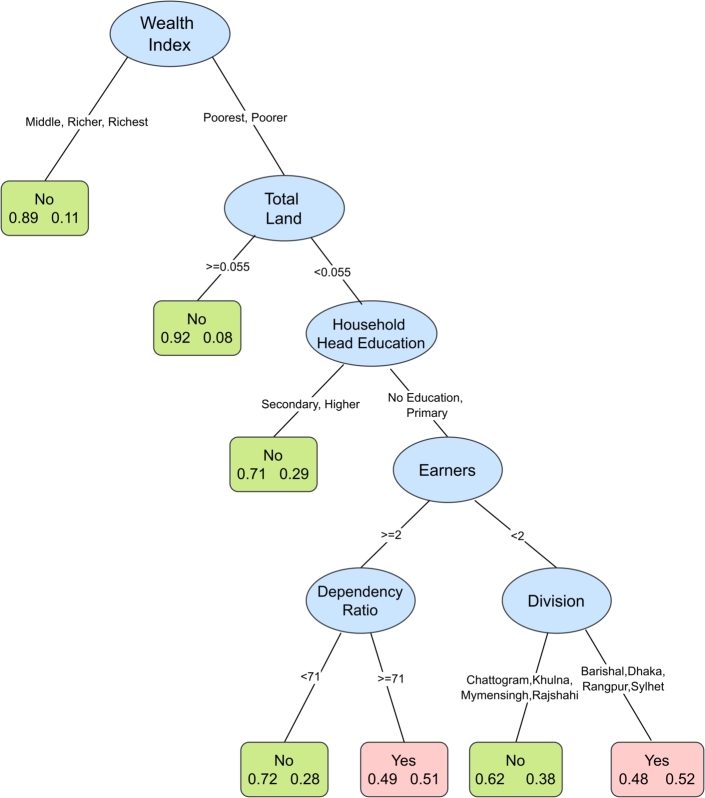
Classification tree for moderate food insecurity. The tree includes factors predicting moderate food insecurity. The higher the variable appears in the tree structure the more statistically important the variable is. Each box at the terminal node contains two numbers: that on the left indicates percentage food secure and that on the right indicates percentage food insecure. If percentage insecure is higher than percentage secure, the group is predicted as food insecure (Yes) and the box is colored pink. Otherwise, it is predicted as food secure and colored green.

Fig. 2 demonstrates the results from the classification tree for moderate food insecurity. Wealth index serves as the first split, being the most influential predictor. Households in the lowest two quintiles are allocated into the right branch, where total land area constitutes the second split in the tree. It reveals the presence of interaction between these two variables. For the households having total land less than 0.055 acres the education level of household heads represents the next split, indicating that it is the third most important variable in predicting moderate level of food insecurity. Other notable predictors are found to be number of earners, administrative division and dependency ratio. This classification tree also helps us to identify the subgroups most vulnerable to moderate food insecurity. Among households in the poorer or poorest group with less than 0.055 acres of total land, a household head with no education or only primary education, two or more earners, and a dependency ratio of 71 or higher are likely to experience moderate food insecurity. When the number of earners is fewer than 2, households located in the division Barishal, Dhaka, Rangpur or Sylhet are likely to belong in the moderate food insecure group.

**Figure 3:**
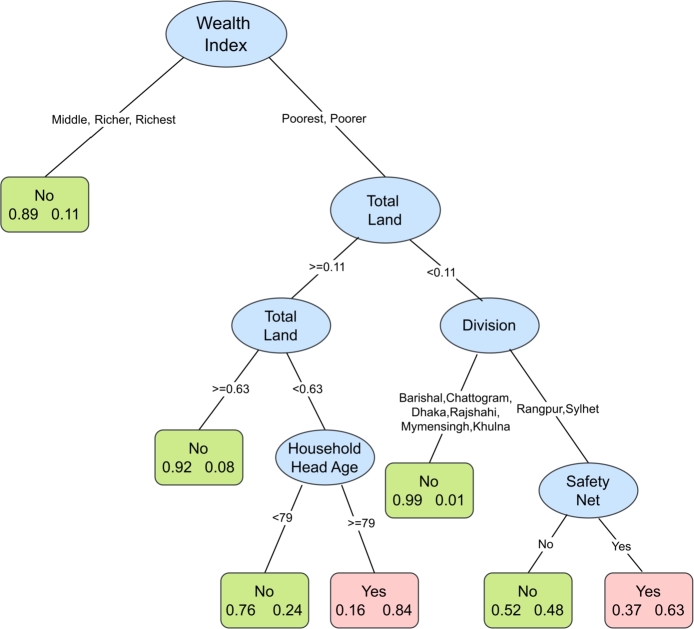
Classification tree for moderate or severe food insecurity. The tree includes factors predicting moderate to severe food insecurity. The higher the variable appears in the tree structure the more statistically important the variable is. Each box at the terminal node contains two numbers: that on the left indicates percentage food secure and that on the right indicates percentage food insecure. If percentage insecure is higher than percentage secure, the group is predicted as food insecure (Yes) and the box is colored pink. Otherwise, it is predicted as food secure and colored green.

Fig. 3 presents the outcomes from the classification tree for moderate or severe food insecurity. Analogous with the case of moderate food insecurity, wealth index is found to be the most important predictor comprising the first split at the root of the tree. Households belonging to the upper three wealth quintiles are not likely to suffer from moderate or severe food insecurity. Poorer and poorest group households comprise the right branch and a further subbranch is created based on total land within this branch, indicating an interaction between these two variables. Households in the lowest 2 wealth quintiles with a total land of 0.11 acres or more and less than 0.63 acres and having household head aged greater or equal to 79 years are likely to experience moderate or severe food insecurity. Households with less than 0.11 acres of total land show a subsequent partition based on their division suggesting a further existence of interaction with the variable division. Specifically, for Rangpur and Sylhet divisions, another factor safety net determines the food insecurity status. If the household receives safety net, they are likely to experience moderate or severe food insecurity.

**Figure 4:**
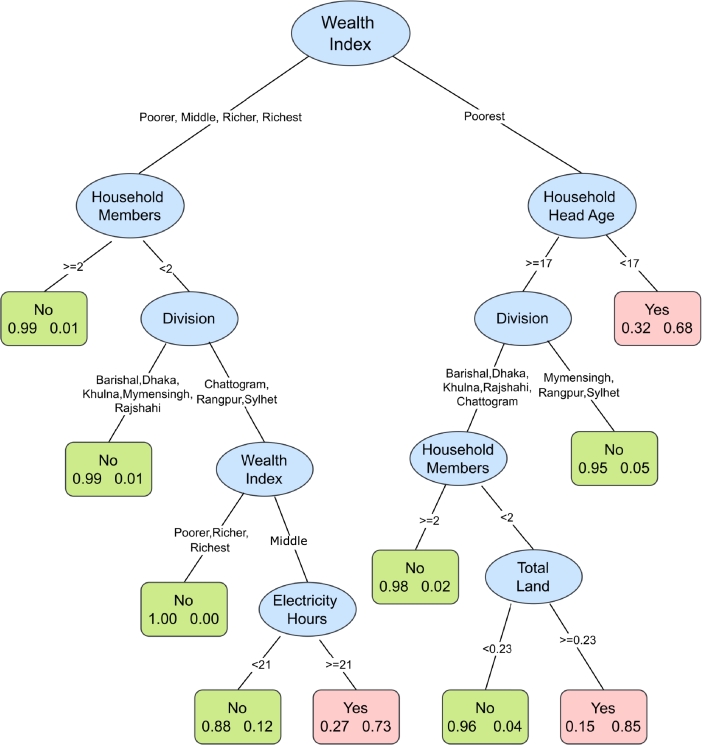
Classification tree for severe food insecurity. The tree includes factors predicting severe food insecurity. The higher the variable appears in the tree structure the more statistically important the variable is. Each box at the terminal node contains two numbers: that on the left indicates percentage food secure and that on the right indicates percentage food insecure. If percentage insecure is higher than percentage secure, the group is predicted as food insecure (Yes) and the box is colored pink. Otherwise, it is predicted as food secure and colored green.

Fig. 4 illustrates the results from the classification tree for severe food insecurity. Similar to moderate or severe and moderate food insecurity, wealth index is the prominent predictor. The upper four wealth quintiles comprises the left branch and the poorest quintile comprises the right branch. In contrast to the previous two levels of food insecurity, the number of household members and the age of the household head are found to play significant roles in predicting severe food insecurity, leading to the second split in the tree. For a household in the poorest wealth quintile with a household head under 17 years of age are likely to fall in the severe food insecure group. Whereas, households in the upper four wealth quintiles with fewer than 2 household members, located in the Chattogram, Rangpur or Sylhet division, belonging to the middle wealth quintile and having access to electricity for 21 hours or more are most likely to belong in the severe food insecure group.

### 4.4 Variable importance plot

**Figure 5:**
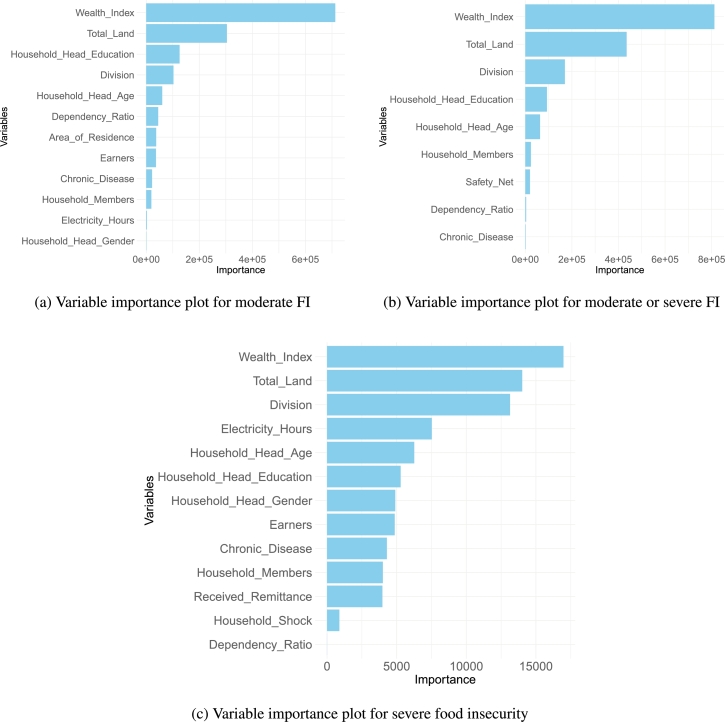
Variable importance plots for three levels of food insecurity: (a) Highlighting key variables contributing to the likelihood of moderate FI. (b) Showing the most significant predictors for moderate or severe FI. (c) Identifying critical variables driving severe FI.

Fig. 5 illustrates the influential predictors for the prediction of three levels of food insecurity. Across all levels, the wealth index emerges as the most significant variable, followed by land ownership. Household head education and division also demonstrate notable importance in predicting food insecurity across these levels. In predicting severe food insecurity specifically, electricity hours, household head age, gender, and number of earners in the household play crucial roles, distinguishing them from their lesser impact on moderate and moderate-to-severe levels of food insecurity. It is noteworthy that the wealth index variable alone predicts moderate or severe and moderate levels of food insecurity quite well. However, in the case of severe food insecurity, variables such as total land and division are nearly equally influential compared to the wealth index.

### 4.5 Multilevel logistic regression model

Table 3 presents the variables associated with different levels of food insecurity from the multilevel logistic regression model, along with the odds ratios and 95% confidence intervals. Households in urban areas with young household heads, owning small quantities of land, receiving safety nets, facing shocks, and residing in the poor wealth class, notably in the Rangpur or Sylhet divisions, are more likely to encounter moderate or moderate-to-severe levels of food insecurity. Additional factors that increase the odds of food insecurity include having an uneducated household head, a high dependency ratio, a low number of household members, few earners, and not receiving remittances. The risk of moderate or moderate-to-severe food insecurity is significantly higher among urban households (*OR* = 1.204, 95% *CI*: 1.010 - 1.436 and *OR* = 1.219, 95% *CI*: 1.020 - 1.457, respectively for moderate and moderate or severe food insecurity) compared to rural households. In addition, for one year increase in household heads age, the odds of facing moderate and moderate or severe food insecurity deceases by 0.8% (*OR* = 0.992, 95% *CI*: 0.987 - 0.996) and 0.7% (*OR* = 0.993, 95% *CI*: 0.989 - 0.997), respectively. The multilevel logistic regression models also indicate that households that own low amount of land are more likely to face moderate and moderate or severe food insecurity (*OR* = 0.800, 95% *CI*: 0.736 - 0.870 and *OR* = 0.770, 95% *CI*: 0.706 - 0.840). Households that received some sort of shock have 35% (*OR* = 1.352, 95% *CI*: 1.168 - 1.566) and 44% (*OR* = 1.443, 95% *CI*: 1.248 - 1.669) higher odds of experiencing moderate and moderate or severe food insecurity, respectively. The risk of both moderate and moderate or severe food insecurity is significantly lower in the poorer, middle, richer, and richest wealth classes compared to the poorest wealth class. There is a noticeable variation in risk of moderate and moderate or severe food insecurity across the eight administrative divisions of Bangladesh: the odds is 60% and 83% higher among the households from Rangpur (*OR* = 1.600, 95% *CI*: 1.149 - 2.228 and *OR* = 1.829, 95% *CI*: 1.306 - 2.562) division compared to the households from Barishal division. Households where the head has received higher education have 74% (*OR* = 0.260, 95% *CI*: 0.206 - 0.329) lower odds of experiencing moderate food insecurity and 75% (*OR* = 0.244, 95% *CI*: 0.194 - 0.308) lower odds of experiencing moderate or severe food insecurity compared to households where the head has no education. Households with a higher number of earners are less likely to experience moderate or moderate-to-severe levels of food insecurity. Specifically, each additional earner in the household is associated with an 18.3% decrease in the odds of facing moderate food insecurity (*OR* = 0.817, 95% *CI*: 0.751 - 0.888) and a similar 18.3% decrease in the odds of facing moderate or severe food insecurity (*OR* = 0.817, 95% *CI*: 0.752 - 0.888). Households that received remittances have significantly lower odds of experiencing moderate (*OR* = 0.346, 95% *CI*: 0.282 - 0.423) and moderate or severe (*OR* = 0.333, 95% *CI*: 0.273 - 0.407) food insecurity compared to those that did not receive remittances.

**Table 3:** Results from multilevel logistic regression to identify the determinants of moderate, moderate or severe and severe food insecurity.

**Variables**	**Moderate FI**		**Moderate or Severe FI**		**Severe FI**	
	**OR (95**% **CI)**	**p-value**	**OR (95**% **CI)**	**p-value**	**OR (95**% **CI)**	**p-value**
* **(Intercept)**	0.860 (0.458, 1.613)	0.638	0.807 (0.429, 1.518)	0.506	0.002 (0.000, 0.015)	<0.001
**Area of Residence**						
Rural						
Urban	1.204 (1.010, 1.436)	0.038	1.219 (1.020, 1.457)	0.029	1.129 (0.713, 1.786)	0.605
**Electricity Hours**	1.015 (0.989, 1.041)	0.264	1.024 (0.998, 1.051)	0.070	1.106 (1.012, 1.209)	0.027
**Household Head Sex**						
Male						
Female	1.100 (0.925, 1.307)	0.281	1.206 (1.018, 1.429)	0.030	1.731 (1.116, 2.684)	0.014
**Household Head Age**	0.992 (0.987, 0.996)	<0.001	0.993 (0.989, 0.997)	0.001	1.006 (0.993, 1.020)	0.334
**Total Land**	0.800 (0.736, 0.870)	<0.001	0.770 (0.706, 0.840)	<0.001	0.103 (0.034, 0.315)	<0.001
**Safety Net**						
No						
Yes	1.472 (1.318, 1.643)	<0.001	1.485 (1.332, 1.657)	<0.001	1.149 (0.813, 1.624)	0.432
**Household Shock**						
No						
Yes	1.352 (1.168, 1.566)	<0.001	1.443 (1.248, 1.669)	<0.001	1.957 (1.322, 2.897)	0.001
**Chronic Disease**						
No						
Yes	1.029 (0.916, 1.156)	0.632	1.042 (0.929, 1.170)	0.483	1.094 (0.761, 1.572)	0.627
**Wealth Index**						
Poorest						
Poorer	0.766 (0.666, 0.881)	<0.001	0.724 (0.630, 0.831)	<0.001	0.701 (0.463, 1.061)	0.093
Middle	0.527 (0.455, 0.611)	<0.001	0.486 (0.420, 0.563)	<0.001	0.475 (0.299, 0.755)	0.002
Richer	0.347 (0.292, 0.413)	<0.001	0.310 (0.261, 0.367)	<0.001	0.265 (0.144, 0.490)	<0.001
Richest	0.130 (0.100, 0.169)	<0.001	0.111 (0.085, 0.144)	<0.001	0.000 (0.000, Inf)	0.868
**Division**						
Barishal						
Chattogram	1.134 (0.801, 1.604)	0.479	1.200 (0.844, 1.708)	0.310	2.283 (0.840, 6.204)	0.106
Dhaka	1.126 (0.801, 1.582)	0.494	1.128 (0.798, 1.595)	0.496	0.903 (0.302, 2.703)	0.855
Khulna	0.863 (0.611, 1.219)	0.404	0.839 (0.590, 1.193)	0.329	0.382 (0.099, 1.474)	0.162
Mymensingh	0.617 (0.438, 0.869)	0.006	0.694 (0.491, 0.982)	0.039	2.395 (0.930, 6.167)	0.070
Rajshahi	0.829 (0.589, 1.166)	0.281	0.834 (0.589, 1.180)	0.306	1.127 (0.410, 3.096)	0.816
Rangpur	1.600 (1.149, 2.228)	0.005	1.829 (1.306, 2.562)	<0.001	4.135 (1.652, 10.348)	0.002
Sylhet	1.353 (0.964, 1.898)	0.081	1.572 (1.115, 2.217)	0.010	4.205 (1.665, 10.616)	0.002
**Household Head Education**						
No Education						
Primary	0.766 (0.673, 0.871)	<0.001	0.725 (0.638, 0.824)	<0.001	0.573 (0.383, 0.856)	0.007
Secondary	0.466 (0.403, 0.539)	<0.001	0.445 (0.385, 0.513)	<0.001	0.571 (0.358, 0.908)	0.018
Higher	0.260 (0.206, 0.329)	<0.001	0.244 (0.194, 0.308)	<0.001	0.269 (0.091, 0.791)	0.017
**Dependency Ratio**	1.002 (1.001, 1.003)	0.001	1.002 (1.001, 1.003)	<0.001	1.001 (0.998, 1.004)	0.589
**Household Members**	0.902 (0.865, 0.940)	<0.001	0.884 (0.849, 0.921)	<0.001	0.843 (0.738, 0.962)	0.011
**Earners**	0.817 (0.751, 0.888)	<0.001	0.817 (0.752, 0.888)	<0.001	0.874 (0.671, 1.140)	0.321
**Received Remittance**						
No						
Yes	0.346 (0.282, 0.423)	<0.001	0.333 (0.273, 0.407)	<0.001	0.561 (0.303, 1.038)	0.066

While several variables are associated with moderate and moderate or severe food insecurity, not all are associated with severe food insecurity. Significant variables include the hours of electricity the household enjoys, the gender of the household head, the amount of land owned, whether the household faced any shocks, wealth index, division, educational attainment of the household head, and the number of household members. Households with female heads have 1.731 times higher odds of experience severe FI compared to households with male heads, with a 95% *CI* ranging from 1.116 to 2.684. While it was observed that the age of household heads was a highly significant variable associated with moderate and moderate or severe food insecurity, its impact on severe food insecurity is not significant. In terms of land ownership, households with larger amounts of land owned have substantially lower odds of experiencing severe FI (*OR* = 0.103, 95% *CI*: 0.034 - 0.315). If a household experienced a shock (compared to those that did not), the odds of severe food insecurity are nearly doubled, with an odds ratio of 1.957 (95% *CI*: 1.322 - 2.897). The odds of severe FI is also more than 4 times higher in Rangpur (*OR* = 4.135, 95% *CI*: 1.625 - 10.348) and Sylhet (*OR* = 4.205, 95% *CI*: 1.665 - 10.616) division compared to Barishal. Having more household members appears to be beneficial in reducing severe food insecurity. Each additional household member is associated with a 16% reduction in the odds of experiencing severe food insecurity (*OR* = 0.843, 95% *CI*: 0.738 - 0.962). Although receiving remittances is not significantly associated with severe food insecurity at the 5% significance level, it nonetheless reduces the odds of severe food insecurity substantially. Specifically, households receiving remittances have 44% lower odds of experiencing severe food insecurity compared to households that did not receive any form of remittances.

To assess the predictive performance of our models, we constructed Receiver Operating Characteristic (ROC) curves for each. The area under the ROC curve (AUC) for the multilevel logistic regression model predicting moderate food insecurity is 0.845, signifying that our model accurately predicts moderate food insecurity for 84% of households. For severe food insecurity, the AUC is 0.959, indicating a high predictive accuracy of 96% for households experiencing severe food insecurity. Similarly, the AUC for predicting moderate or severe food insecurity is 0.853, highlighting an 85% accuracy in identifying households facing either moderate or severe food insecurity.

### 4.6 Choropleth map of food insecurity

**Figure 6:**
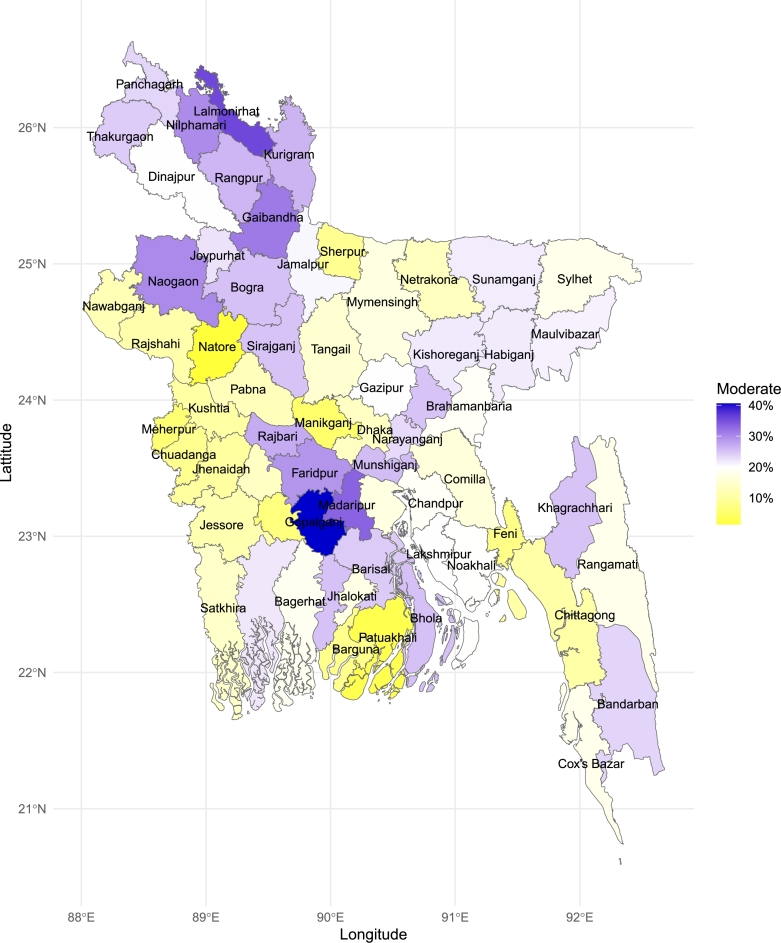
Prevalence of moderate food insecurity across districts in Bangladesh: The choropleth map highlights the geographic distribution of moderate food insecurity, identifying districts with higher prevalence.

**Figure 7:**
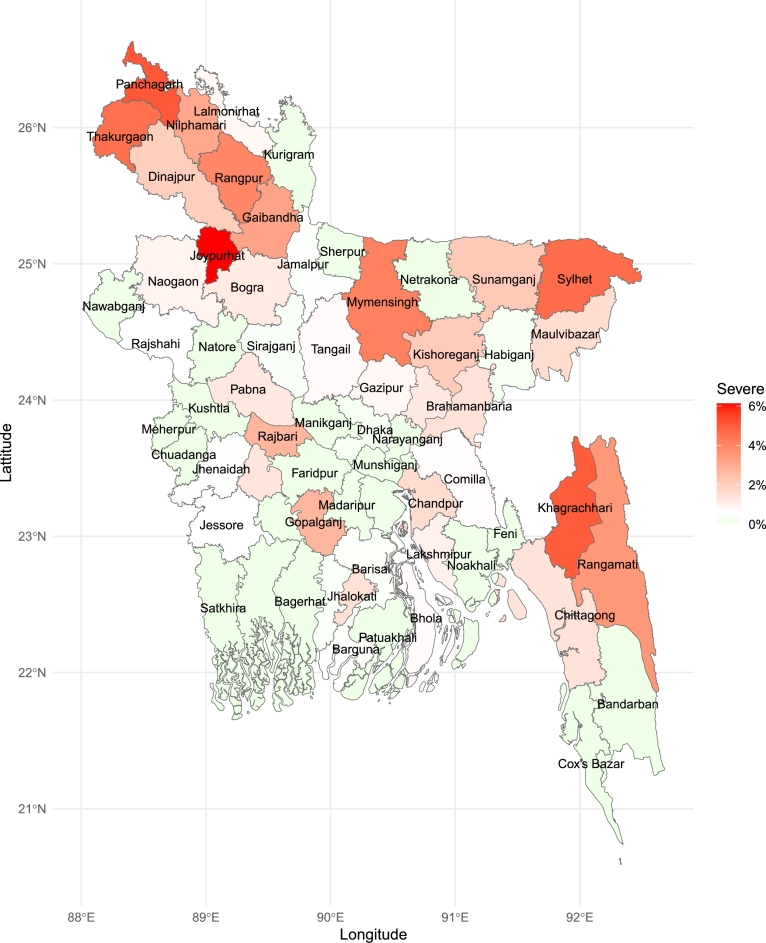
Prevalence of severe food insecurity across districts in Bangladesh: The choropleth map highlights the geographic distribution of severe food insecurity, identifying districts with higher prevalence.

We have conducted district-wise maps of the prevalence of moderate food insecurity and severe food insecurity. As shown in Fig. 6, moderate food insecurity is notably more prevalent in the northern districts. Gopalganj district demonstrates the highest prevalence of moderate food insecurity, with rates approaching 40%. In contrast, the districts of Natore and Patuakhali have the lowest prevalence of moderate food insecurity, with rates significantly below 10%. Fig. 7 reveals that severe food insecurity is particularly high in districts bordering both India and Myanmar, with significant concentrations in the southeastern hill tract regions, a pattern not observed for moderate food insecurity. Joypurhat district has the highest prevalence of severe food insecurity, while most districts in the northern region have a prevalence rate exceeding 4%. Both moderate and severe food insecurity prevalences are low in the western districts of the country. The coastal districts also display a notably low prevalence of both moderate and severe food insecurity indicating a relative stability in food security compared to other regions.

## 5 Discussion

This study utilized data from the HIES 2022, which, for the first time, collected information on food insecurity using the Food Insecurity Experience Scale (FIES) developed by the FAO [22]. FIES is a globally recognized tool to measure food insecurity through individuals' and households' direct experiences. Unlike traditional methods that rely on metrics such as food expenditure, food diversity, and calorie intake - widely used in Bangladesh to measure food scarcity (e.g., [48], [25]) - FIES captures the psychological and behavioral dimensions of food insecurity through survey-based questions, addressing concerns like food supply anxiety, reduced food intake, and experiences of hunger.

In a developing country like Bangladesh, a strong link between food insecurity and household wealth is expected, and our study confirms this relationship. Limited financial resources restrict access to adequate nutrition, meaning wealthier households can afford diverse and nutritious foods, while poorer households often rely on less nutritious, cheaper staples. The prevalence of high food insecurity among lower wealth quintiles in Bangladesh has been widely documented [32], [26]. A closely related finding in our study is the significant association between food insecurity and land ownership. Agricultural productivity, which is closely tied to land ownership, can influence food security, especially in rural areas. Households that own land or assets can produce their own food, reducing dependence on volatile market prices. This connection between land entitlement and food insecurity has been established in several other studies [32].

Education within a household also emerged as a crucial determinant of food insecurity in our study, consistent with existing literature [32], [49]. Households with higher levels of education, particularly that of the household head, generally experience lower levels of food insecurity. Education enhances employment opportunities and income potential, improving access to sufficient and nutritious food. Additionally, education provides individuals with better knowledge about nutrition and health, enabling informed decisions regarding food purchases and preparation. This is especially important in rural areas where food variety and availability are limited. Education also fosters better financial management skills, helping households budget and save for lean periods, thereby reducing their vulnerability to food shortages.

Our study further found that exposure to shocks is positively associated with food insecurity. Shocks such as natural disasters, agricultural failures, and economic downturns (e.g., unemployment and inflation) can strain household resources, limiting their ability to purchase sufficient and nutritious food, and exacerbating food insecurity among vulnerable populations [50], [51]. Given Bangladesh's vulnerability to cyclones, floods, and riverbank erosion, these findings align with previous research that has documented the impact of such events on food insecurity [52], [53].

Another key finding is the relationship between remittance inflows and lower food insecurity. In Bangladesh, remittances from migrant workers play a crucial role in influencing household food security, particularly in rural areas. These financial inflows significantly enhance household purchasing power, enabling access to a more diverse and nutritious diet. Previous studies have shown that households receiving remittances are less likely to experience food insecurity compared to non-recipients, as remittances help smooth consumption and mitigate the impacts of local economic shocks [54], [29]. Our findings corroborate the strong association between remittance earnings and improved food security.

Additionally, our study examined the spatial variation in the prevalence of food insecurity, revealing that the northern districts of Bangladesh, specially the land-locked, nearby boarder areas, hilly areas with low waterbody experienced the highest levels of moderate and severe food insecurity. This finding is consistent with earlier research on seasonal food insecurity, which identified similar trends [15], [42]. The high prevalence of food insecurity in the northwestern districts also aligns with studies that suggest these regions are hotspots of food insecurity [40]. The identified regions have low per capita arable land and a low productivity of staple crops, and poor irrigation system [16], [55]. Furthermore, administrative divisions like Rangpur and Sylhet, and the hill-tracts area that accommodate several severely food insecure districts largely have lower per capita income and lower literacy [16]. Conversely, district-wise maps indicate a lower prevalence of food insecurity in coastal areas, supporting the positive impact of climate-smart agricultural practices promoted by the Bangladesh Ministry of Agriculture in those regions [56].

A unique contribution of our study is the exploration of interactions between potential predictors of food insecurity using classification trees. We observed that households at the highest risk of food insecurity are often identified by the interplay of multiple factors rather than a single factor in isolation. For instance, a combination of low wealth, limited land ownership, low education levels, and high dependency ratios significantly increases a household's vulnerability to food insecurity. Although the application of machine learning for food security in Bangladesh remains relatively limited, studies conducted in other countries demonstrate the significant predictive power of these methods, reinforcing the validity of using machine learning tree-based approaches in this context [57], [58]. In our study, the same set of covariates emerges as significant factors influencing food security in tree-based and regression modeling approaches. Although there has been no prior research in Bangladesh that directly compares machine learning and regression models within the context of food security, analogous studies have been conducted in relation to public health issues [59]. The maps showing district-level food insecurity prevalence provide a better understanding of regional variations and highlight the need for district-specific interventions to address areas with high food insecurity prevalence.

## 6 Conclusion

Bangladesh has experienced significant economic growth and poverty reduction, yet food insecurity remains a concern. Based on the Household Income and Expenditure Survey 2022, about 20.49% of households are moderately to severely food insecure. Key factors influencing food security include wealth, land ownership, education, family size, remittance income, and crisis exposure. Policy implications emphasize the promotion of education, family planning, employment opportunities, and remittance inflows, particularly in rural areas. Strategies should focus on targeting high-risk districts with interventions addressing multiple factors simultaneously, such as financial incentives for family planning and nutrition education.

## Ethical statement

The authors did not collect any data but rather used secondary sources. Household Income and Expenditure Survey (HIES) 2022 is carried out by Bangladesh Bureau of Statistics (BBS) under the guidance and technical support from the World Bank Group. An oral consent was taken from each of the respondents before the interview. All the respondents are unidentified.

## CRediT authorship contribution statement

**Faria Rauf Ria:** Writing – original draft, Methodology, Formal analysis, Data curation, Conceptualization. **Md. Muhitul Alam:** Writing – original draft, Methodology, Formal analysis, Conceptualization. **Md. Azad Uddin:** Writing – original draft, Methodology, Conceptualization. **Mohaimen Mansur:** Writing – original draft, Methodology, Conceptualization. **Md. Israt Rayhan:** Writing – review & editing, Writing – original draft, Supervision.

## Declaration of Competing Interest

The authors declare that they have no known competing financial interests or personal relationships that could have appeared to influence the work reported in this paper.

## Data Availability

This paper uses nationally representative data from the Household Income and Expenditure Survey (HIES) 2022 conducted by Bangladesh Bureau of Statistics. The data are available upon request from Bangladesh Bureau of Statistics (BBS) website at https://bbs.gov.bd/.
